# An Exploratory Pilot Study of Inflammatory Cytokine Gene Polymorphisms and Residual Postoperative Outcomes in Older Women One Year After Bariatric Surgery

**DOI:** 10.3390/nu18081294

**Published:** 2026-04-20

**Authors:** Dante Mafra Tourino Teixeira, Antonio Avelino Ferreira Soares, Renata de Souza Freitas, Larissa Sousa Silva Bonasser, Caroline Ferreira Fratelli, Calliandra Maria de Souza Silva, Evelyn Mikaela Kogawa, Linconl Agudo Oliveira Benito, Izabel Cristina Rodrigues da Silva

**Affiliations:** 1 Postgraduate Program in Health Sciences and Technologies, Faculty of Health Sciences and Technology, University of Brasilia (UnB), Brasilia-Federal District (DF), Brasilia 72220-900, Brazil; mafra.dante11@gmail.com (D.M.T.T.); aavelinofs@gmail.com (A.A.F.S.); carolfratelli@gmail.com (C.F.F.); cdssilva@gmail.com (C.M.d.S.S.); 2 Postgraduate Program in Clinical Psychology and Culture, Institute of Psychology, University of Brasilia (UnB), Brasília-Federal District (DF), Brasilia 72220-900, Brazil; laribonasser@gmail.com; 3 Academic Unit of Biotechnology Engineering (UAEB), Center for Sustainable Development of the Semi-Arid Region (CDSA), Sumé Campus, Federal University of Campina Grande, Sumé-Paraíba, Sumé 58540-000, Brazil; 4 Department of Dentistry, School of Health Sciences, University of Brasilia (UnB), Brasilia-Federal District (DF), Brasilia 70910-900, Brazil; mikaela.kogawa@unb.br; 5 Nursing Department, Centro Universitário de Brasilia, Asa Norte Campus, Brasilia-Federal District (DF), Brasilia 70790-075, Brazil; linconlbenito@yahoo.com.br

**Keywords:** bariatric surgery, obesity, polymorphism, genetic, inflammation, aged

## Abstract

Background/Objectives: Obesity is characterized by chronic low-grade inflammation, and bariatric surgery promotes substantial metabolic and inflammatory improvement. However, residual obesity and microvascular complications may persist in some individuals, suggesting potential genetic influences on postoperative outcomes. This exploratory pilot study investigated the association between inflammatory cytokine gene polymorphisms and clinical, metabolic, and inflammatory outcomes in older women one year after bariatric surgery. Methods: This cross-sectional, hypothesis-generating pilot study included 21 women aged ≥50 years (mean 61.6 ± 5.0) who underwent Roux-en-Y gastric bypass at a public bariatric center in Brazil. Anthropometry, body composition, biochemical markers, and serum levels of interleukin-6 (IL-6) and tumor necrosis factor-α (TNF-α) were assessed 12 months postoperatively. Genotyping for *IL6*-174G/C (rs1800795) and *TNFA*-308G/A (rs1800629) was performed using PCR-RFLP. Associations were analyzed using non-parametric statistical tests. Results: Notably, the *IL6*-174CC genotype was associated with persistent obesity, whereas carriers of the *TNFA*-308A allele showed a higher prevalence of diabetic retinopathy. These results highlight genotype-specific postoperative outcomes. No significant genotype-related differences were observed for most anthropometric, biochemical, or inflammatory parameters, indicating substantial overall metabolic improvement after surgery regardless of genetic background. However, the observed associations were based on a small sample and should be interpreted cautiously. Conclusions: This exploratory pilot study revealed associations between inflammatory cytokine gene polymorphisms and selected postoperative outcomes, particularly persistent obesity and diabetic retinopathy, in older women one year after bariatric surgery. These hypothesis-generating findings emphasize the need for larger, longitudinal studies to clarify the role of genetic factors in postoperative heterogeneity after bariatric surgery.

## 1. Introduction

Obesity is a chronic, multifactorial condition characterized by abnormal lipid accumulation and excessive adipose tissue, which contributes to a persistent low-grade systemic inflammation [1,2]. This inflammatory milieu contributes to the development of insulin resistance, type 2 diabetes mellitus, cardiovascular disease, and other metabolic complications, disproportionately affecting older adults due to age-related metabolic and physiological changes [3,4]. As global population aging progresses, obesity in elderly individuals has emerged as a growing public health concern, with substantial clinical and economic implications [5,6].

Bariatric surgery is currently recognized as the most effective therapeutic strategy for sustained weight loss and improvement of obesity-related comorbidities [7,8]. Beyond mechanical restriction and nutrient malabsorption, procedures such as Roux-en-Y gastric bypass (RYGB) induce profound metabolic and immunological remodeling, leading to reductions in systemic inflammation and improvement of glycemic and lipid profiles [9,10]. Consequently, bariatric surgery has increasingly been conceptualized as “metabolic surgery” [11]. Although these benefits are well established, postoperative outcomes are heterogeneous, and a subset of patients continues to exhibit residual obesity or obesity-related complications despite adequate surgical intervention [12].

In older populations, this heterogeneity is further accentuated. Aging is associated with reductions in basal metabolic rate, loss of lean mass, altered adipose tissue distribution, and impaired inflammatory resolution, potentially limiting the magnitude or durability of postoperative metabolic improvement [4]. Despite growing evidence supporting the safety and effectiveness of bariatric surgery in older adults, age-related physiological changes may interact with inflammatory pathways, influencing long-term outcomes [12,13].

Patients’ genetic background represents a potential contributor to this variability. Polymorphisms in genes encoding pro-inflammatory cytokines may influence cytokine expression, inflammatory tone, and tissue responses to metabolic stress [14,15]. Among these, interleukin-6 (IL-6) and tumor necrosis factor-α (TNF-α) play central roles in obesity-related inflammation, insulin resistance, endothelial dysfunction, and microvascular injury [3,16]. The *IL6*-174G/C (rs1800795) polymorphism has been associated with differential transcriptional activity and circulating IL-6 levels, while the *TNFA*-308G/A (rs1800629) polymorphism has been linked to increased TNF-α expression and susceptibility to metabolic and vascular complications [16,17,18].

However, evidence regarding the influence of these polymorphisms on postoperative outcomes after bariatric surgery remains limited and inconsistent. Most previous studies have focused on younger or mixed-age populations and have primarily evaluated short-term metabolic endpoints, with limited attention to inflammatory resolution or microvascular outcomes in older adults [7,14]. Data specifically addressing whether inflammatory gene polymorphisms modulate residual obesity or microvascular complications in older women after bariatric surgery are scarce.

Despite the well-established metabolic and inflammatory benefits of bariatric surgery, substantial interindividual variability in postoperative outcomes persists, particularly among older adults. Age-related metabolic alterations, prolong exposure to obesity-associated inflammation, and reduced physiological resilience may contribute to heterogeneous responses, including persistent obesity and microvascular complications.

Although inflammatory cytokines such as interleukin-6 (IL-6) and tumor necrosis factor-α (TNF-α) are central to obesity-related inflammation and vascular dysfunction, evidence on whether genetic polymorphisms in these pathways affect postoperative outcomes after bariatric surgery remains limited, particularly in older women. Most available studies have focused on younger or mixed-age populations and have seldom addressed residual clinical outcomes in older women.

Therefore, this exploratory, hypothesis-generating pilot study investigated associations between the *IL6*-174G/C (rs1800795) and *TNFA*-308G/A (rs1800629) polymorphisms and postoperative anthropometric, biochemical, inflammatory, and clinical outcomes in older women one year after Roux-en-Y gastric bypass, with particular attention to persistent obesity and microvascular complications.

## 2. Materials and Methods

### 2.1. Study Design and Participants

This study was designed as a cross-sectional, exploratory, and analytical investigation, conducted 12 months after bariatric surgery. The objective was to explore associations between inflammatory cytokine gene polymorphisms and postoperative anthropometric, biochemical, inflammatory, and clinical outcomes. Due to its cross-sectional design, this study does not allow for causal inference or for assessing temporal changes following surgery.

The study population comprised 21 women aged ≥50 years (mean 61.6 ± 5.0 years) who had undergone Roux-en-Y gastric bypass (RYGB) exactly one year prior to data collection. All participants were recruited from the bariatric surgery reference service of the Federal District’s State Health Department (SESDF), Brazil, a public tertiary center operating under standardized multidisciplinary postoperative follow-up protocols.

Given its exploratory design and the limited sample size, this study was not intended to establish causal relationships or definitive genetic associations but rather to generate hypotheses regarding potential genotype–phenotype relationships in this specific population.

### 2.2. Eligibility Criteria

Inclusion criteria were: (i) female sex; (ii) age ≥50 years at the time of evaluation; (iii) completion of at least 12 months of postoperative follow-up after RYGB; (iv) bariatric surgery performed at the Regional Hospital of Asa Norte (HRAN); and (v) cognitive ability to understand and respond to study procedures.

Exclusion criteria included: (i) age < 50 years; (ii) bariatric surgery performed less than 12 months prior to evaluation; (iii) bariatric procedures performed at other institutions; and (iv) documented psychiatric or neurological conditions that could compromise informed consent or data reliability. No participants reported diagnosed autoimmune or chronic inflammatory diseases or use of immunosuppressive therapy at the time of evaluation; however, residual confounding by unmeasured inflammatory conditions cannot be entirely excluded.

### 2.3. Ethical Considerations

The study was conducted in accordance with the Declaration of Helsinki and approved by the Research Ethics Committee of the Health Sciences Teaching and Research Foundation (FEPECS), State Secretariat of Health of the Federal District (approval number 1.910.166). All participants provided written informed consent prior to participation. 

### 2.4. Clinical and Anthropometric Assessment

Clinical and anthropometric data were obtained during routine postoperative follow-up visits and from medical records. Trained healthcare professionals measured body weight and height, and body mass index (BMI) was calculated as weight (kg)/height^2^ (m^2^).

Persistent obesity was operationally defined as BMI ≥ 30 kg/m^2^ at 12 months after surgery, in accordance with World Health Organization criteria. Obesity-related clinical conditions (e.g., hypertension, dyslipidemia, neuropathy) were identified from documented medical diagnoses in patient records.

Preoperative anthropometric and metabolic data were not available for comparison, as the present analysis focused exclusively on postoperative outcomes at 12 months. Therefore, changes over time could not be assessed.

Diabetic retinopathy was considered present when recorded in the medical chart by an ophthalmologist during routine diabetes follow-up; no additional ophthalmological examinations were conducted specifically for this study.

### 2.5. Clinical and Laboratory Evaluation

Blood samples were collected 12 months postoperatively following an overnight fast. All biochemical analyses—including fasting blood glucose, lipid profile (total cholesterol, HDL-C, LDL-C, VLDL-C, triglycerides), and total lipid concentration—were performed in the same certified laboratory using standardized commercial assays.

Serum total vitamin D [25-hydroxyvitamin D, 25(OH)D] concentrations were measured using a chemiluminescence immunoassay (LIAISON^®^ 25(OH)D Total Assay, DiaSorin, Via Crescentino s.n.c., 13040-Saluggia, Vercelli, Italy), with a functional sensitivity of <10 nmol/L and an inter-assay coefficient of variation of 7.8%.

Serum concentrations of interleukin-6 (IL-6) and tumor necrosis factor-α (TNF-α) were determined using enzyme-linked immunosorbent assay (ELISA) kits specific for human cytokines (Invitrogen/Thermo Fisher Scientific, 81 Wyman Street, Waltham, MA, USA). The minimum detectable concentrations were 1.1 pg/mL for IL-6 and 4.8 pg/mL for TNF-α. Cytokine measurements were obtained at a single time point; therefore, intraindividual variability over time could not be assessed.

Information regarding diabetes duration, historical glycemic control (e.g., HbA1c), detailed dietary intake, physical activity levels, and medication use was not systematically available and therefore could not be included in the analyses.

### 2.6. Body Composition Assessment

Body composition was assessed using dual-energy X-ray absorptiometry (DXA) (Lunar Prodigy Advance, GE Healthcare, 500 West Monroe Street, Chicago, IL, USA). Measurements of fat mass, lean mass, and total body bone mineral density were performed by the same trained operator. Participants were instructed to fast for at least 4 h and to avoid physical activity for 24 h before the exam. The DXA device was calibrated before each measurement session. The coefficients of variation in the laboratory were 1.03% for fat mass, 1.35% for lean mass, and 0.83% for bone mineral density.

### 2.7. Genotyping

Genomic DNA was extracted from peripheral blood samples using standard procedures. Genotyping of *IL6*-174G/C (rs1800795) polymorphism was performed using polymerase chain reaction (PCR) combined with restriction fragment length polymorphism (RFLP) analysis [19]. The forward and reverse primers used to amplify *IL6*-174G/C (rs1800795) were 5′-ATG CCA AAG TGC TGA GTC ACT A-3′ and 5′-GGA AAA TCC CAC ATT TGA TA-3′, respectively. The cycling program used for amplification was as follows: an initial denaturing step of 5 min at 94 °C and subsequently 35 denaturing cycles of 1 min at 94 °C, annealing of 1 min at 55 °C and extension of 50 s at 72 °C, ending with a final extension of 7 min at 72 °C. The PCR product was subjected to RFLP analysis with the restriction enzyme *Nla*III (New England BioLabs Inc., Ipswich, MA, USA). The resulting product should consist of 232 base pairs in the absence of polymorphism (GG), while the presence (CC) should result in two bands, one band of 125 base pairs and another of 107 base pairs. PCR-RFLP products were visualized in a 3% agarose gel, stained with ethidium bromide, and exposed to ultraviolet light.

The *TNFA*-308G/A (rs1800629) polymorphism was also genotyped using PCR-RFLP [20]. The forward and reverse primers used to amplify *TNFA*-308G/A (rs1800629) were 5′-AGG CAA TAG GTT TTG AGG GCC AT-3′ and 5′-TCC TCC CTG CTC CGA TTC CG-3′, respectively. The amplification was performed using the following cycle program: an initial denaturing step at 95 °C for 10 min, then 38 denaturing cycles at 94 °C for 1 min, annealing at 57 °C for 1 min and extension to 72 °C for 1 min, followed by a final extension at 72 °C for 8 min. 107 bp PCR products were subsequently incubated for 90 min at 37 °C with the enzyme *Nco*I. The allele G was cut into two fragments of 87 and 20 bp, while allele A remained undigested. These fragments were visualized in a 3.5% agarose gel stained with ethidium bromide and exposed to ultraviolet light.

Genotype distributions were evaluated within a phenotype-enriched clinical sample. For the *IL6*-174G/C polymorphism, GG and GC genotypes were combined in subsequent analyses due to the low frequency of heterozygotes and to improve analytical stability in this exploratory study.

### 2.8. Statistical Analysis

Clinical characteristics were expressed as categorical variables or genotypic frequencies. Differences between *TNFA*-308G/A and *IL6*-174G/C genotypes and anthropometric, biochemical, and immunological parameters were evaluated using non-parametric tests, with results presented as quartiles. Hardy–Weinberg equilibrium was assessed using the chi-square test (*df* = 1) based on observed genotype frequencies in the study sample. Analyses were performed using SPSS version 29.0 (SPSS Inc., Chicago, IL, USA), with a significance level of 5%.

Given the small sample size and exploratory nature of the study, all statistical analyses were conducted with the primary aim of identifying preliminary associations rather than establishing definitive genetic effects. Therefore, findings should be interpreted with caution and regarded as hypothesis-generating.

The sample size (n = 21) was estimated using a 95% confidence interval, a 15% margin of error, a population size of 28, and a response rate of 50%, yielding a minimum of 18 participants after accounting for a 15% loss rate. It is acknowledged that this sample size is limited for formal genetic association studies and may increase the risk of type I error. Accordingly, the present analyses were designed to be exploratory and descriptive in nature.

## 3. Results

### 3.1. TNFA-308 G/A Polymorphism

A comprehensive analysis of anthropometric, biochemical, and clinical variables according to the *TNFA*-308G/A polymorphism is presented in Table 1 and Table 2. TNF-α serum concentrations did not differ significantly between individuals with the GG genotype and those heterozygous for AG (*p* = 0.485). Percentile distributions of TNF-α values (P25, median, P75) showed substantial overlap between genotypes, indicating that the presence of the A allele did not influence circulating TNF-α levels in this cohort. This lack of genotypic effect was consistent across all additional biochemical markers assessed, including fasting blood glucose, total cholesterol, LDL-C, HDL-C, VLDL, triglycerides, non-HDL cholesterol, vitamin D, and total lipid concentration. For all parameters, *p*-values exceeded the conventional threshold (*p* > 0.05), indicating a homogeneous metabolic profile across genotype.

Similarly, body composition metrics obtained by densitometric analysis—namely, total fat mass, lean mass, and total bone mineral density—showed no significant differences between individuals with the GG genotype and those with the AG genotype. Percentile distributions once again exhibited substantial overlap, reinforcing the interpretation that the *TNFA*-308G/A polymorphism does not exert a significant influence on adiposity, musculoskeletal composition, or bone density in this clinical sample.

Table 2 summarizes the distribution of qualitative and clinical variables. Across multiple comorbidities and symptoms—including hypertension, depression/anxiety, fibromyalgia, dyslipidemia, vaginal dryness, history of hyperosmolar coma, gastrointestinal symptoms (dysphagia or dyspepsia), neuropathy, amputation, and musculoskeletal pain (arthralgia or myalgia)—no significant associations with genotype were observed (all *p* > 0.05). These findings suggest that the *TNFA*-308G/A variant does not influence the expression of these clinical conditions in individuals undergoing bariatric surgery.

The sole statistically significant association identified was with diabetic retinopathy. Carriers of the *TNFA*-308 A allele showed a higher prevalence of retinopathy compared to GG genotype carriers (*p* = 0.027). This result is based on a limited number of cases and should therefore be interpreted cautiously, particularly given the exploratory nature of the study and the absence of longitudinal clinical data.

Genotypic frequency analysis revealed that 61.9% of participants carried the GG genotype and 38.1% the AG genotype, while the AA genotype was absent, consistent with a low minor allele frequency. The distribution conformed to Hardy–Weinberg equilibrium (*p* = 0.281), indicating no evidence of selective pressure, nonrandom mating, or mutational distortion at this locus.

### 3.2. IL6-174G/C Polymorphism

Table 3 displays biochemical and anthropometric variables stratified by *IL6*-174 G/C genotype. Median IL-6 serum concentrations did not differ significantly between GG+GC carriers and CC homozygotes (*p* = 0.240). Although CC homozygotes exhibited a broader interquartile range, the lack of statistical significance suggests that IL-6 level variability was not attributable to this polymorphism. Similarly, no significant differences were observed for fasting blood glucose, total cholesterol, triglycerides, LDL-C, HDL-C, VLDL, non-HDL cholesterol, total lipids, vitamin D levels, and densitometric markers (fat mass, lean mass, and bone mineral density), with all *p* > 0.05. These findings collectively indicate that this IL6 gene variant does not contribute to interindividual variability in biochemical or anthropometric parameters in this cohort.

Table 4 outlines the distribution of qualitative variables by genotype. As with the *TNFA* polymorphism, most clinical conditions—including hypertension, depression/anxiety, fibromyalgia, dyslipidemia, hyperosmolar coma, vaginal dryness, retinopathy, nephropathy, neuropathy, amputation, musculoskeletal pain, and gastrointestinal symptoms—showed no significant associations (all *p* > 0.05), suggesting that the IL6 promoter variant is not an essential determinant of these clinical features within this cohort.

A notable exception was observed for obesity. A statistically significant association was found between the *IL6*-174 CC genotype and persistent obesity at 12 months post-surgery (*p* = 0.035). However, this association was based on a very small number of cases, and no significant differences were detected for continuous anthropometric, biochemical, or inflammatory variables across genotypes.

Genotypic frequencies were 47.62% for GG, 19.05% for GC, and 33.33% for CC. Genotypic distribution for the *IL6*-174G/C polymorphism deviated from Hardy–Weinberg equilibrium (*p* = 0.005), which is likely attributable to the phenotype-enriched nature of the sample, consisting exclusively of older women with a history of severe obesity undergoing bariatric surgery, rather than to genotyping error.

Overall, postoperative anthropometric, biochemical, and inflammatory profiles improved substantially across genotypes. Apart from the associations described above, no consistent genotype-related differences were observed, reinforcing the exploratory and descriptive nature of these findings.

## 4. Discussion

This exploratory pilot study investigated whether polymorphisms in inflammatory cytokine genes (*IL6*-174G/C and *TNFA*-308G/A) are associated with postoperative anthropometric, inflammatory, and clinical outcomes in older women one year after Roux-en-Y gastric bypass. Two preliminary genotype–phenotype associations were observed: persistent obesity among carriers of the *IL6*-174 CC genotype and a higher prevalence of diabetic retinopathy among carriers of the *TNFA*-308 A allele. In contrast, most anthropometric, biochemical, and inflammatory parameters demonstrated substantial postoperative improvement irrespective of genotype [8,10].

Given the cross-sectional design, small sample size, and reliance on single-time-point inflammatory measurements, these findings should be interpreted cautiously and regarded as hypothesis-generating rather than confirmatory.

### 4.1. IL6-174G/C Polymorphism and Obesity Persistence

The association between the *IL6*-174 CC genotype and persistent obesity observed in this study should be interpreted with caution. Although the C allele has been characterized as a low-producing IL6 variant in functional studies, circulating IL-6 concentrations did not differ significantly between genotypes in the present sample [21,22]. This apparent discrepancy may reflect the dominant anti-inflammatory effects of bariatric surgery, age-related alterations in inflammatory regulation, and the limitation of single postoperative cytokine measurements [23].

Importantly, the observed association was based on a very small number of CC carriers with persistent obesity, and no significant genotype-related differences were detected for continuous anthropometric or body composition variables. Therefore, this finding should be considered preliminary and may reflect residual variability in metabolic adaptation rather than a direct genetic effect [2,3].

### 4.2. TNFA-308G/A Polymorphism and Retinopathy

The *TNFA*-308G/A polymorphism was not associated with postoperative metabolic parameters or circulating TNF-α levels in this cohort. Carriers of the *TNFA*-308 A allele exhibited a higher prevalence of diabetic retinopathy in this cohort. This finding is biologically plausible, as the −308 A allele has been associated with increased TNF-α transcriptional activity and susceptibility to microvascular complications in previous studies. However, the present analysis did not assess the onset, progression, or regression of retinopathy following bariatric surgery [17,24,25].

Consequently, this association most likely reflects the persistence of pre-existing microvascular damage rather than an insufficient metabolic response to surgery. Chronic inflammatory exposure and endothelial dysfunction may result in irreversible structural changes that are not fully reversed despite substantial postoperative metabolic improvement [3,26].

### 4.3. Genotype-Independent Metabolic Improvements

Despite genotype-specific associations, the majority of anthropometric, biochemical, and body composition parameters improved substantially across all genotypes. These findings are consistent with previous evidence demonstrating that bariatric surgery induces broad metabolic and inflammatory benefits, largely independent of genetic background [7,11]. Long-term reductions in low-grade systemic inflammation after surgery have been consistently reported, although not all inflammatory mediators normalize uniformly [8,10].

The lack of significant differences in circulating IL-6 and TNF-α levels across genotypes may also be explained by the profound systemic anti-inflammatory effects induced by bariatric surgery. In older individuals, postoperative inflammatory profiles may be more strongly influenced by surgical, metabolic, and age-related factors than by modest genetic variation. Furthermore, circulating cytokine concentrations may not adequately reflect tissue-specific inflammatory signaling relevant to adipose tissue or microvascular compartments.

### 4.4. Clinical Implications and Future Directions

Although the present findings do not support a major role for inflammatory gene polymorphisms in determining overall metabolic response to bariatric surgery, they suggest that genetic background may contribute to residual heterogeneity in specific postoperative outcomes, such as persistent obesity or microvascular complications. In older women—who may have longer exposure to metabolic stress and reduced physiological resilience—such genetic influences may have implications for individualized postoperative monitoring strategies [12,13].

Future studies should include larger, more diverse populations, longitudinal inflammatory profiling, and adjustment for key clinical confounders, such as obesity duration, diabetes history, glycemic control, and lifestyle factors. Integration of genetic data with epigenetic and transcriptomic analyses may further elucidate mechanisms underlying postoperative heterogeneity and support the development of more personalized postoperative care strategies [14,15].

Several limitations should be acknowledged. The small sample size limits statistical power and increases the risk of type I error, precluding definitive genetic association inferences. The cross-sectional design and absence of preoperative data or a non-surgical control group prevent assessment of temporal changes and causal relationships. Additionally, information on diabetes duration, historical glycemic control, lifestyle factors, medication use, and potential age-related confounders was unavailable.

Deviations from the Hardy–Weinberg equilibrium for the IL6 polymorphism are likely due to the phenotype-enriched nature of this bariatric cohort rather than genotyping error. Collectively, these limitations reinforce the exploratory character of the study and underscore the need for cautious interpretation.

Despite these limitations, the present findings suggest that host genetic background may contribute to residual heterogeneity in selected postoperative outcomes after bariatric surgery, particularly in older women with long-standing metabolic burden. Rather than supporting immediate clinical application, these results highlight the importance of further longitudinal studies integrating genetic, inflammatory, and clinical data to clarify mechanisms underlying postoperative variability and to inform personalized postoperative monitoring strategies.

## 5. Conclusions

This exploratory pilot study evaluated associations between inflammatory cytokine gene polymorphisms and postoperative outcomes in older women one year after Roux-en-Y gastric bypass. While substantial metabolic, anthropometric, and inflammatory improvements were observed across the cohort irrespective of genotype, two preliminary genotype–phenotype associations were identified: persistent obesity among carriers of the *IL6*-174 CC genotype and a higher prevalence of diabetic retinopathy among carriers of the *TNFA*-308 A allele.

Given the cross-sectional design, small sample size, and absence of preoperative data, these findings should be considered hypothesis-generating and interpreted with caution. They do not support causal inference or immediate clinical application, but rather highlight the potential contribution of host genetic background to postoperative heterogeneity in selected outcomes, particularly in older women with long-standing metabolic burden.

Future studies with larger samples, longitudinal designs, and integrated clinical and inflammatory profiling are warranted to confirm these associations and to clarify the role of inflammatory gene polymorphisms in shaping long-term outcomes after bariatric surgery.

## Figures and Tables

**Table 1:** Anthropometric and biochemical characteristics according to *TNFA*-308G/A genotype.

	*TNFA-308G/A*						
	GG (N = 13)			AG (N = 8)			
	P25	Median	P75	P25	Median	P75	*p*
[TNF-α] pg/mL	10.35	13.88	17.89	13.58	15.92	17.44	0.485
Fasting Blood Glucose	84.00	90.50	97.00	74.00	76.50	93.00	0.180
Total Cholesterol	154.00	179.00	200.00	187.00	202.00	212.00	0.240
Triglycerides	96.00	107.50	135.00	91.00	110.00	130.00	0.589
HDL	59.00	61.00	63.00	56.00	60.50	84.00	0.818
LDL	71.80	94.50	106.60	85.40	115.00	129.40	0.394
VLDL	19.20	21.50	27.00	18.20	22.00	26.00	0.589
Non-HDL Cholesterol	91.00	116.00	151.00	119.00	134.50	148.00	0.485
Total lipids	481.00	555.00	635.00	597.50	617.50	643.00	0.394
Vitamin D	19.00	30.50	39.70	23.70	26.75	30.60	0.818
Fat Mass (g)	23,724.00	28,165.00	33,423.00	28,298.00	34,242.50	47,495.50	0.185
Lean Mass (g)	39,428.00	41,134.00	42,962.00	41,535.50	43,100.50	47,274.50	0.076
Total bone mineral density (g)	1903.00	1990.00	2370.00	1987.00	2112.50	2354.00	0.645

**Table 2:** Symptoms, habits and other clinical variables according to the *TNFA*-308G/A genotype.

		*TNFA*-308G/A				
		GG		AG		
		N	%	N	%	*p*
Hypertension	Yes	10	76.9%	8	100.0%	0.142
	No	3	23.1%	0	0.0%	
Depression/anxiety	Yes	5	38.5%	3	37.5%	0.965
	No	8	61.5%	5	62.5%	
Fibromyalgia	Yes	2	15.4%	1	12.5%	0.854
	No	11	84.6%	7	87.5%	
Dyslipidemia	Yes	11	84.6%	1	12.5%	0.854
	No	2	15.4%	7	87.5%	
Hyperosmolar coma	Yes	1	7.7%	0	0.0%	0.421
	No	12	92.3%	8	100.0%	
Vaginal dryness	Yes	3	23.1%	2	25.0%	0.920
	No	10	76.9%	6	75.0%	
Obesity	Yes	1	7.7%	1	12.5%	0.716
	No	12	92.3%	7	87.5%	
Retinopathy	Yes	1	7.7%	4	50.0%	0.027 *
	No	12	92.3%	4	50.0%	
Nephropathy	Yes	0	0.0%	0	0.0%	0.646
	No	13	100.0%	8	100.0%	
Neuropathy	Yes	1	7.7%	0	0.0%	0.421
	No	12	92.3%	8	100.0%	
Amputation	Yes	1	7.7%	0	0.0%	0.421
	No	12	92.3%	8	100.0%	
Arthralgia or myalgia	Yes	3	23.1%	4	50.0%	0.204
	No	10	76.9%	4	50.0%	
Dysphagia or dyspepsia	Yes	2	15.4%	0	0.0%	0.243
	No	11	94.6%	8	100.0%	

* *p* < 0.05.

**Table 3:** Anthropometric and biochemical characteristics according to *IL6*-174G/C genotype.

	*IL6*-174 G/C						
	GG+GC (N = 14)			CC (N= 7)			
	P25	Median	P75	P25	Median	P75	*p*
[IL-6] pg/mL	11.16	12.37	13.68	12.27	16.37	33.67	0.240
Fasting blood glucose	74.00	79.50	84.00	88.00	93.00	97.00	0.093
Total Cholesterol	180.00	191.50	200.00	162.00	202.00	212.00	0.699
Triglycerides	96.00	132.50	193.00	91.00	98.00	113.00	0.180
HDL	51.00	58.50	63.00	59.00	62.50	67.00	0.240
LDL	77.00	96.00	129.40	82.60	103.60	129.20	0.999
VLDL	19.20	26.50	38.60	18.20	19.60	22.60	0.180
Non-HDL Cholesterol	103.00	134.50	151.00	103.00	124.00	148.00	0.818
Total lipids	568.00	616.25	643.00	507.00	607.00	643.00	0.699
Vitamin D	23.70	30.50	35.00	19.00	26.75	30.60	0.589
Fat Mass (g)	26,751.00	30,814.50	33,596.00	20,971.00	27,737.00	42,248.00	0.856
Lean Mass (g)	39,766.00	41,642.50	42,962.00	40,920.00	42,991.00	47,910.00	0.224
Total bone mineral density (g)	1947.00	2005.50	2336.00	1903.00	2364.00	2372.00	0.636

**Table 4:** Symptoms, habits and other clinical variables according to the *IL6*-174G/C genotype.

		*IL6-174 G/C*				
		GG+GC		CC		
		N	%	N	%	*p*
Hypertension	Yes	11	78.6%	7	100.0%	0.186
	No	3	21.4%	0	0.0%	
Depression/anxiety	Yes	4	28.6%	4	57.1%	0.204
	No	10	71.4%	3	42.9%	
Fibromyalgia	Yes	2	14.3%	1	14.3%	0.999
	No	12	85.7%	6	85.7%	
Dyslipidemia	Yes	1	7.1%	2	28.6%	0.186
	No	13	92.9%	5	71.4%	
Hyperosmolar coma	Yes	0	0.0%	1	14.3%	0.147
	No	14	100.0%	6	85.7%	
Vaginal dryness	Yes	2	14.3%	3	42.9%	0.182
	No	12	85.7%	4	57.1%	
Obesity	Yes	0	0.0%	2	28.6%	0.035 *
	No	14	100.0%	5	71.4%	
Retinopathy	Yes	4	28.6%	1	14.3%	0.469
	No	10	71.4%	6	85.7%	
Nephropathy	Yes	0	0.0%	0	0.0%	n/a ^#^
	No	14	100.0%	7	100.0%	
Neuropathy	Yes	1	7.1%	0	0.0%	0.469
	No	13	92.9%	7	100.0%	
Amputation	Yes	0	0.0%	1	14.3%	0.147
	No	14	100.0%	6	85.7%	
Arthralgia or myalgia	Yes	5	35.7%	2	28.6%	0.743
	No	9	64.3%	5	71.4%	
Dysphagia or dyspepsia	Yes	2	14.3%	0	0.0%	0.293
	No	12	85.7%	7	100.0%	

^#^ not applicable. * *p* < 0.05

## Data Availability

The research data are contained in the article’s tables.
